# Supplementary figures and images for: Machine learning-based pipeline for automated intracerebral hemorrhage and drain detection, quantification, and classification in non-enhanced CT images (NeuroDrAIn)
Source: PLoS One. 2024 Dec 26;19(12):e0316003. doi: 10.1371/journal.pone.0316003 (PMC11670976; doi:10.1371/journal.pone.0316003)

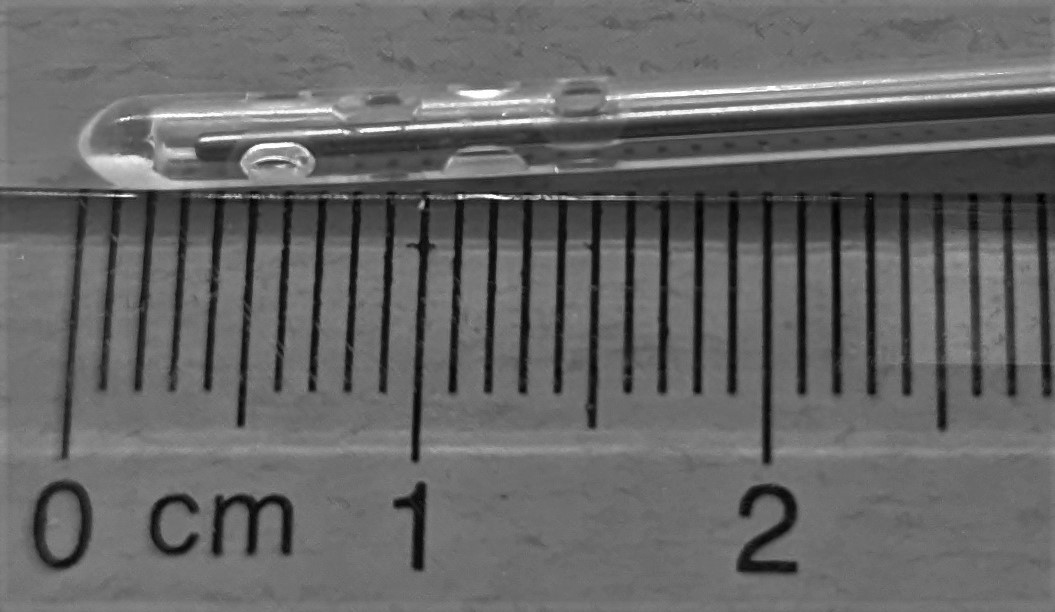

Supplement: S1 Fig — Scale is given in cm. (TIF) [file pone.0316003.s001.tif]

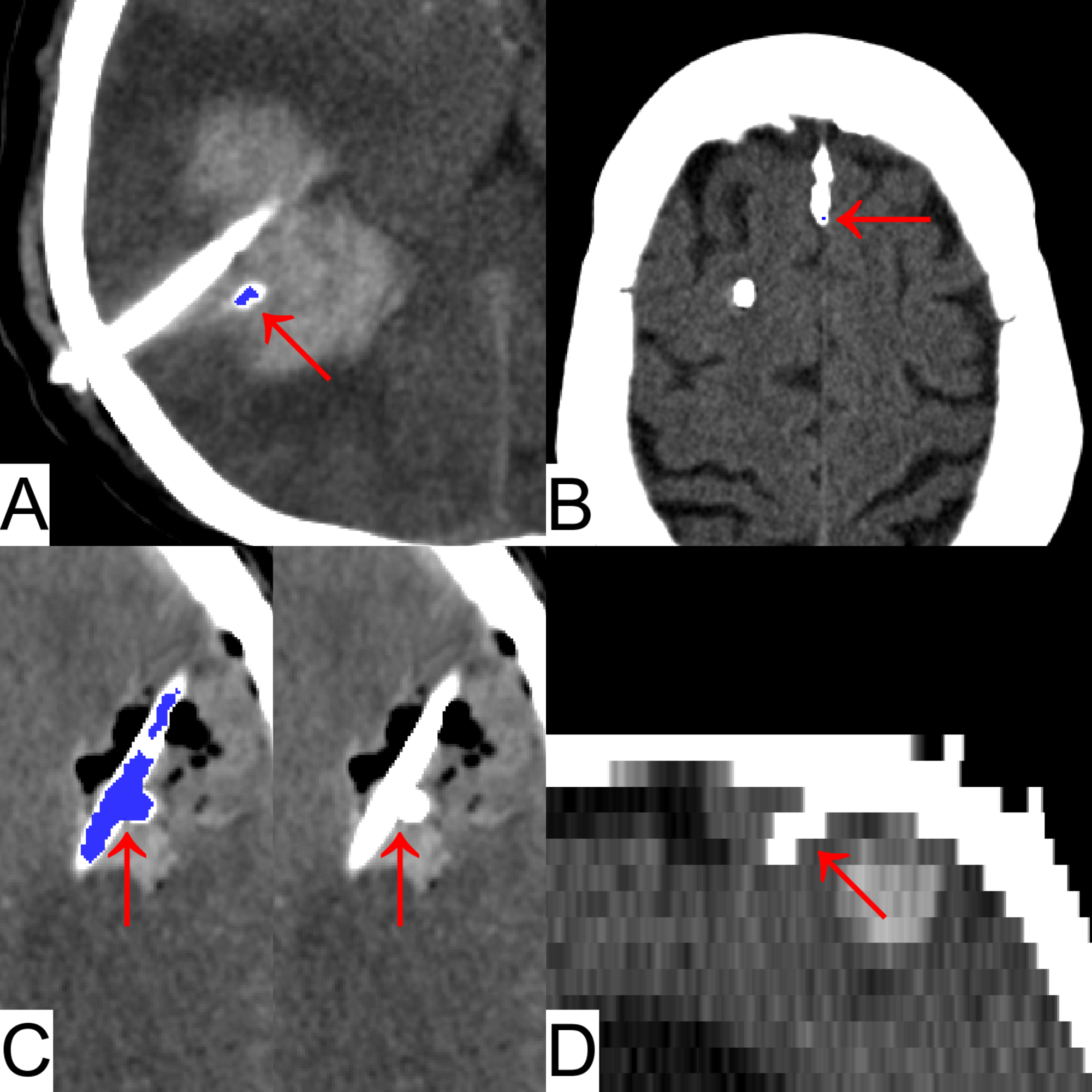

Supplement: S2 Fig — Axial (A-C) and sagittal (D) reconstructions of non-contrast CT scans of falsely detected drains superimposed with the binary drain mask at 0.90 probability threshold (blue). (A) A displaced bone fragment within the bleeding (arrow) and (B) a thick calcification of the flax cerebri (arrow) classified incorrectly as drains. (C) Two touching drains (arrow) segmented as a single object. (D) A drain with a short intracranial course (arrow), missed in the 0.9 threshold binary mask. (TIF) [file pone.0316003.s002.tif]

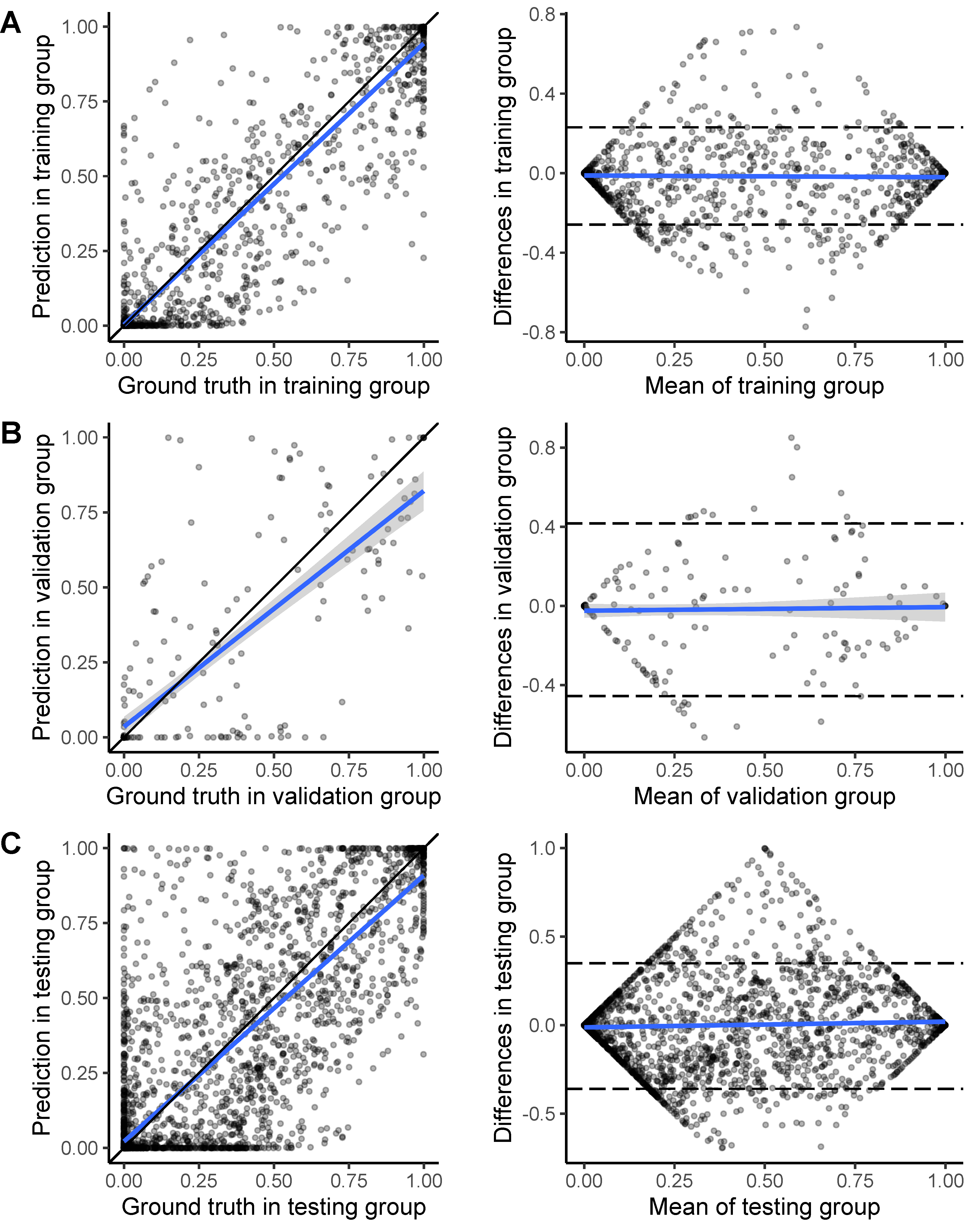

Supplement: S3 Fig — Concordance (left column) and Bland-Altman plots (right column) of the coverage profiles of predicted and GT drains in (A) training, (B) validation and (C) testing datasets. Regression line (blue) and 95% confidence interval of predicted values (shaded area). (TIF) [file pone.0316003.s003.tif]

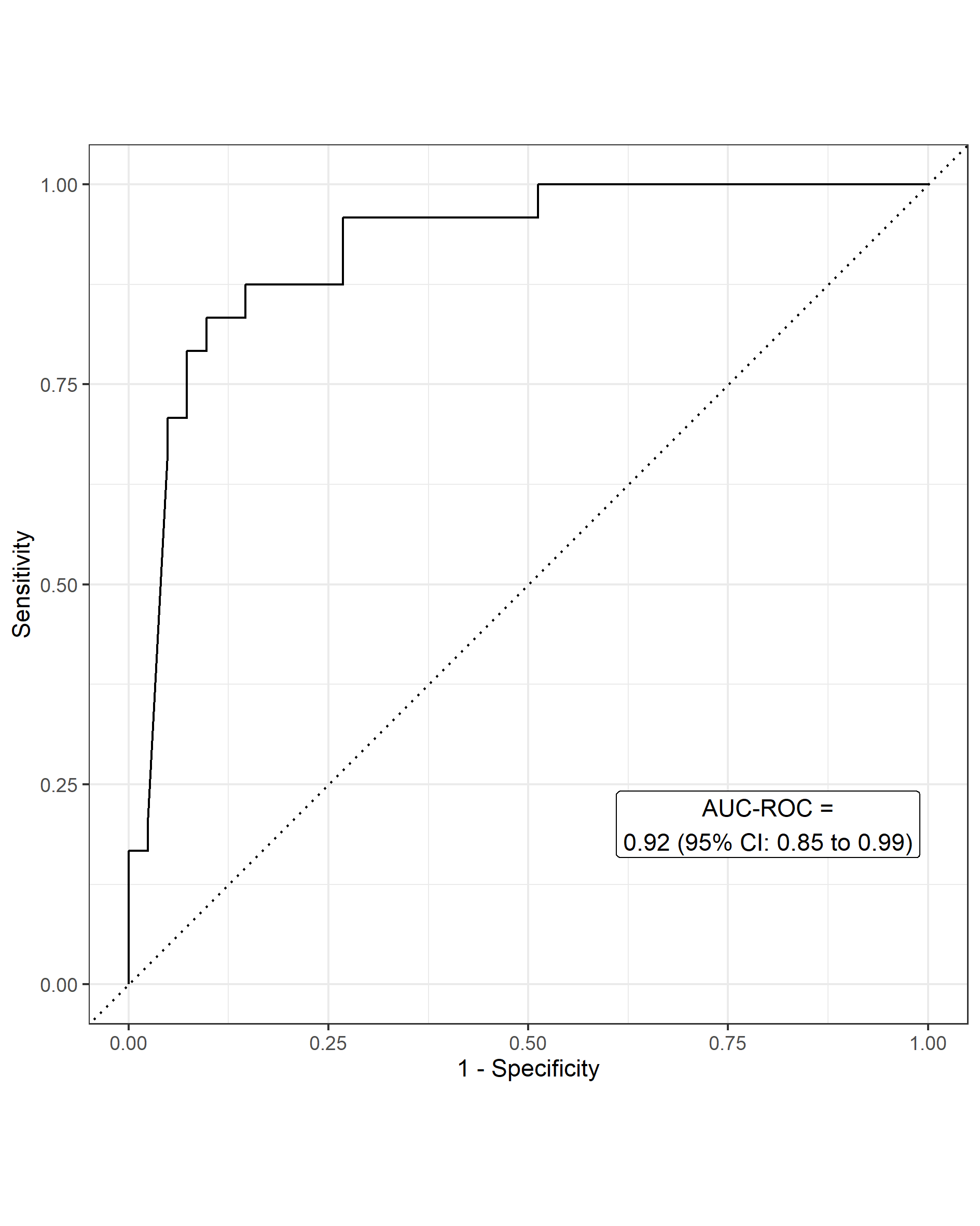

Supplement: S4 Fig — 95% CI: 95% confidence interval. (TIF) [file pone.0316003.s004.tif]

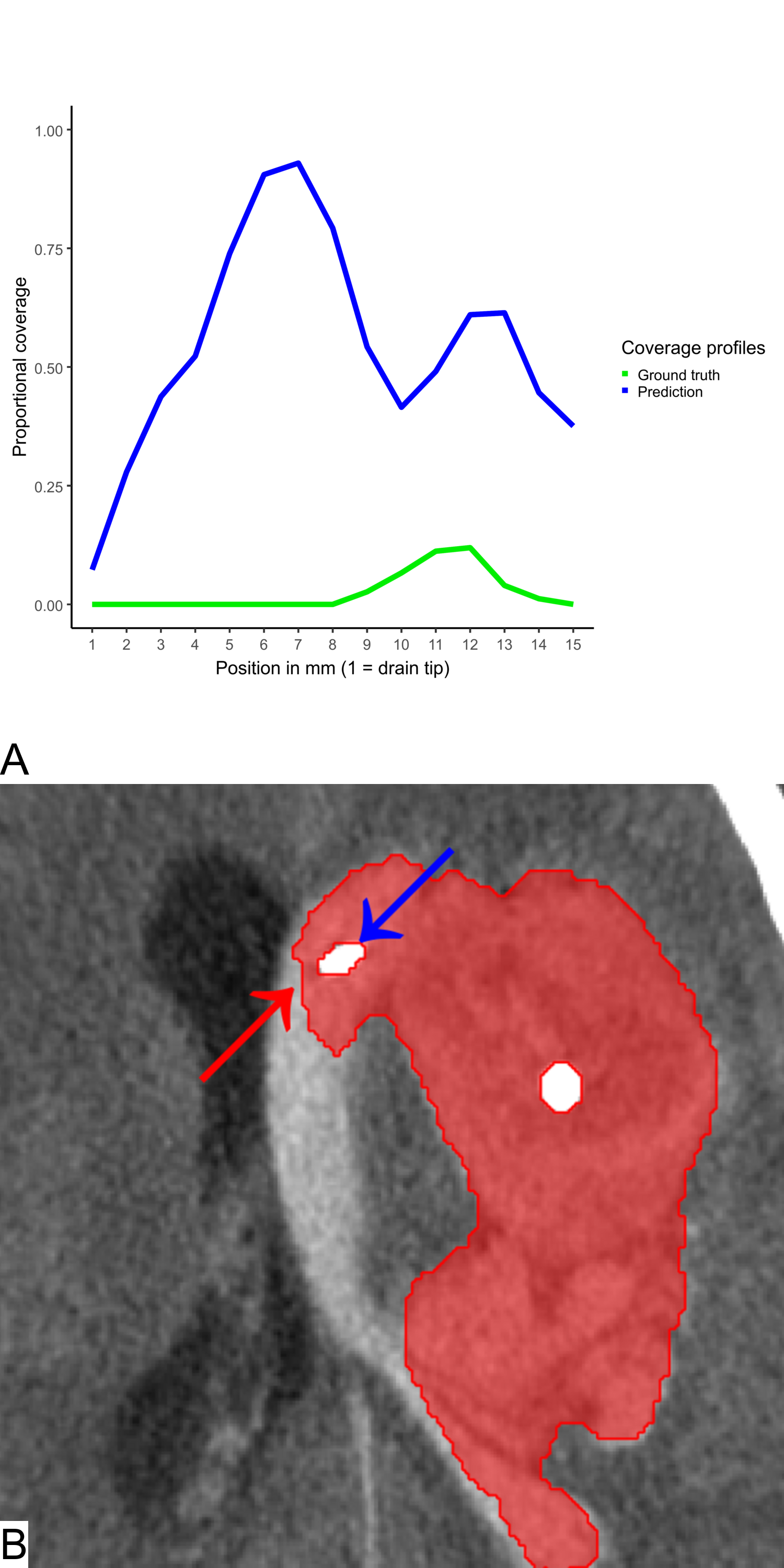

Supplement: S5 Fig — Image depicting (A) predicted (blue) and ground truth (green) coverage profiles of a false positive classification result (drain #8). (B) Corresponding CT scan, showing contact between tip of the external ventricular drain (blue arrow) and an intraventricular bleeding (red arrow). (TIF) [file pone.0316003.s005.tif]
